# Scale and Scope of Gene-Alcohol Interactions in Chronic Pancreatitis: A Systematic Review

**DOI:** 10.3390/genes12040471

**Published:** 2021-03-25

**Authors:** Jian-Min Chen, Anthony F. Herzig, Emmanuelle Génin, Emmanuelle Masson, David N. Cooper, Claude Férec

**Affiliations:** 1 EFS, Univ Brest, Inserm, UMR 1078, GGB, F-29200 Brest, France; anthony.herzig@inserm.fr (A.F.H.); emmanuelle.genin@inserm.fr (E.G.); claude.ferec@univ-brest.fr (C.F.); 2 Service de Génétique Médicale et de Biologie de la Reproduction, CHRU Brest, F-29200 Brest, France; emmanuelle.masson@univ-brest.fr; 3 Institute of Medical Genetics, School of Medicine, Cardiff University, Cardiff CF10 3XQ, UK; CooperDN@cardiff.ac.uk

**Keywords:** genetic predisposition to disease, gene dosage effect, genetic variation, genome-wide association study, human

## Abstract

Background: Excessive alcohol consumption has long been known to be the primary cause of chronic pancreatitis (CP) but genetic risk factors have been increasingly identified over the past 25 years. The scale and scope of gene-alcohol interactions in CP nevertheless remain unclear. Methods: All studies that had obtained genetic variant data concurrently on alcoholic CP (ACP) patients, non-ACP (NACP) patients and normal controls were collated. Employing normal controls as a common baseline, paired OR_ACP_ and OR_NACP_ (odds ratios associated with ACP and NACP, respectively) values were calculated and used to assess gene-alcohol interactions. Results: Thirteen variants involving *PRSS1*, *SPINK1*, *CTRC*, *CLDN2*, *CPA1*, *CEL* and *CTRB1-CTRB2*, and varying from very rare to common, were collated. Seven variants had an OR_ACP_ > OR_NACP_, which was regarded as an immediate indicator of gene-alcohol interactions in CP. Variants with an OR_ACP_ < OR_NACP_ were also found to interact with alcohol consumption by virtue of their impact on age at first pancreatitis symptoms in ACP. Conclusions: This study revealed evidence for extensive gene-alcohol interactions in CP. Our findings lend support to the hypothesis that alcohol affects the expression of genetically determined CP and highlight a predominant role of weak-effect variants in the development of ACP.

## 1. Introduction

Although excessive alcohol consumption has long been known to be the primary cause of chronic pancreatitis (CP), its contribution to the etiology and development of pancreatitis is still shrouded in mystery in many respects [1,2,3,4]. Since the discovery of the first CP-causing variant, namely c.365G>A (p.Arg122His) in the *PRSS1* gene (MIM# 276000; encoding cationic trypsinogen) [5], one new puzzle has emerged pertaining to whether or how a particular genetic risk factor interacts with alcohol consumption to cause CP. For example, one of the most extensively studied genetic risk factors—c.101A>G (p.Asn34Ser) in the *SPINK1* gene (MIM# 167790; encoding pancreatic-specific trypsin inhibitor (PSTI)) [6]—was found to be overrepresented in alcoholic CP (ACP) patients; however, its detection rate in ACP patients was actually lower than that in non-ACP (NACP) patients [7,8,9], making it difficult to discern any gene-environment interactions (G×E) that might exist. Similar situations include, among others, a much lower detection rate of rare pathogenic *CTRC* (MIM# 601405; encoding chymotrypsin C) or *CPA1* (MIM# 114850; encoding carboxypeptidase A1) variants in ACP patients than in NACP patients [10,11].

An interaction between alcohol consumption and the rs10273639-tagging common *PRSS1-PRSS2* haplotype had however been suggested, firstly by the observation that the risk rs10273639C allele was found more frequently in ACP patients than in NACP patients and secondly by a case-only (i.e., ACP vs. NACP) analysis [12]. Recently, by fitting a more sophisticated model and by combining data from several studies, we provided compelling evidence for a synergistic interaction between the common *PRSS1-PRSS2* haplotype and alcohol consumption [13]. *PRSS1* was the first gene discovered to be responsible for CP [5]. Most pathogenic *PRSS1* variants cause or predispose to CP by promoting increased activation or expression of trypsinogen [14], as exemplified by the p.Arg122His missense variant and trypsinogen gene duplication and triplication copy number variants [5,15,16]. *PRSS2* encodes anionic trypsinogen (MIM# 601564), the second major trypsinogen isoform after cationic trypsinogen. Multiple lines of evidence support the involvement of *PRSS2* in CP (see Herzig et al. [13] and references therein), although no pathogenic missense variants in the gene have been reported to date [14].

In addition to gain-of-function *PRSS1* missense and copy number variants, loss-of-function variants in the *SPINK1* gene [6] and *CTRC* gene [10,17] also predispose to CP by bringing about a gain of trypsin activity. Specifically, loss-of-function *SPINK1* variants exert their effect by reducing the capability of PSTI-trypsin binding (see Szabo et al. [18] and references therein) whereas most loss-of-function *CTRC* variants increase trypsin activity by impairing (protective) trypsinogen degradation [10,19]. Together, genetic studies of the three genes have led to the identification of a trypsin-dependent pathological pathway in CP [20].

There is a pronounced dosage effect of the aforementioned rs10273639C risk allele on *PRSS1/PRSS2* mRNA expression in human pancreatic tissue [12,13]. Given that uncontrolled trypsin expression/activity is central to CP pathogenesis [5,12,15,21,22,23,24,25], the synergistic interaction revealed between the common *PRSS1-PRSS2* haplotype and alcohol consumption [13] may serve as an important reference for unravelling the underlying mechanisms responsible for the G×E interactions in CP.

The three trypsin-dependent pathway genes (*PRSS1, SPINK1* and *CTRC*) are among the most extensively studied CP genes in the context of NACP as well as ACP. We propose that a systematic review and meta-analysis of the currently available genetic data, starting from a comparison of the risk (in terms of odds ratio (OR)) conferred upon ACP (termed OR_ACP_) by known pathogenic variants in the trypsin-dependent pathway genes (as well as other CP susceptibility genes) with the corresponding risk conferred to NACP (termed OR_NACP_), could shed new light on the scale and scope of G×E interactions in CP. This study represents just such an attempt.

## 2. Materials and Methods

### 2.1. Disease Definitions

ACP was defined here as in the original publications despite slight differences in terms of the amount and duration of alcohol consumed as well as the ethnicity of the studied cohorts. For example, in most studies, ACP was attributed in relation to an alcohol intake of ≥80 g/d for a male and 60 g/d for a female for at least two years although both amount and duration of alcohol consumption were not specified in some studies. NACP and idiopathic chronic pancreatitis (ICP) were used here interchangeably. In principle, NACP was diagnosed in CP patients who were not known to have a positive family history and whose disease was not caused by known etiologies such as heavy drinking, autoimmune or obstructive factors.

### 2.2. Research Strategy

**Figure 1:**
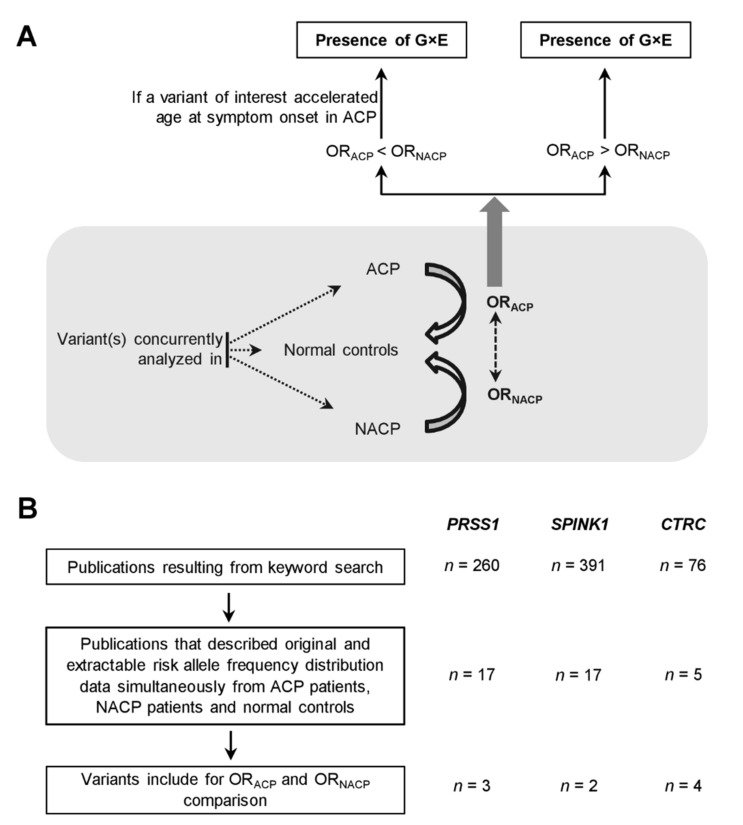
(**A**) Outline of the research strategy. (**B**) Flow chart of the search and selection process for the studies used for the analysis in the context of the three trypsin-dependent pathway genes. ACP, alcoholic chronic pancreatitis; G×E, gene-environment interaction; NACP, non-alcoholic chronic pancreatitis; OR_ACP_, odds ratio associated with ACP; OR_NACP_, odds ratio associated with NACP.

The outline of the research strategy is provided in Figure 1A. The key step was to generate paired OR_ACP_ and OR_NACP_ values for the previously reported variants in the three trypsin-dependent pathway genes as well as several other CP susceptibility genes. This was achieved through the re-analysis of available studies that had obtained variant data concurrently on ACP patients, NACP patients and normal controls, with normal controls being employed as a common baseline to calculate the respective ORs. In parallel to this main analysis, and in the context of the three trypsin-dependent pathway genes, we also compared the risk allele frequencies of informative variants in simultaneously analyzed normal and alcoholic controls. Alcoholic controls denote either subjects with alcohol dependence but without chronic pancreatitis or subjects with alcohol-associated liver cirrhosis. Normal controls denote healthy or population controls.

The resulting paired OR_ACP_ and OR_NACP_ values were used firstly to evaluate the scale of G×E interactions in CP. Thus, if a variant had an OR_ACP_ > OR_NACP_, it was interpreted as denoting an interaction with alcohol consumption. When variants exhibited an OR_ACP_ < OR_NACP_, a further literature search was performed with a view to establishing their impact on the age at symptom onset in ACP: if a given variant was shown to accelerate age at symptom onset in variant-positive ACP patients compared to variant-negative ACP patients, this was interpreted as denoting an interaction with alcohol consumption.

### 2.3. Literature Searches and Selection Processes

Literature searches, performed with the intention of generating paired OR_ACP_ and OR_NACP_ values, in the context of the *PRSS1*, *SPINK1* and *CTRC* genes, respectively, employed keyword queries in “all fields” of PubMed, from August 2020 to November 2020 and were frozen on 20 November 2020. For each gene, different sets of keyword query were first performed, with the results from the different queries being combined to generate a single list of unique publications (for details, see Supplementary Figure S1). All keyword search-derived publications were manually reviewed; only those that described original and extractable risk allele frequency distribution data simultaneously from ACP patients, NACP patients and normal controls were retained for analysis. In cases of overlapping informative studies from the same group, it was the latest one that was used for analysis. In the context of additional CP genes, only the first disease association report that included variant data from ACP, NACP and normal controls was used for analysis whenever possible; subsequent studies were briefly discussed regarding confirmation (or otherwise) of the first association report.

### 2.4. Data Extraction

All relevant data were manually extracted from the included studies.

### 2.5. Statistical Analysis

For a given variant (or aggregated variants), and in the case of single study-derived data, the OR_ACP_ or OR_NACP_, associated 95% confidence interval (CI), and related χ^2^ test of significance of the difference between risk allele frequencies, were calculated from the appropriate 2-by-2 contingency table in R [26]. A difference was regarded as being statistically significant when the *p* value was ≤0.05. If, for a given variant, data were available from ≥2 studies, the variant was subject to meta-analysis with respect to the calculation of its OR, 95% CI and *p* values. Meta-analysis, heterogeneity analysis, forest plots and funnel plots were performed using Review Manager 5.3 software [27]. The Mantel–Haenszel fixed-effect model was used to compute the pooled OR in the absence of statistical heterogeneity; otherwise, the Mantel–Haenszel random-effect model was used. Heterogeneity was considered to be significant when the *p* value for the test of heterogeneity was <0.05 or *I*^2^ was 50% or more [27,28]. Funnel plots were performed only when the number of included studies that generated estimable ORs was ≥10 [27,29].

This study essentially followed PRISMA (Preferred Reporting Items for Systematic Reviews and Meta-Analyses) guidelines [30]. The study was not registered in PROSPERO (an international database of prospectively registered systematic reviews), which does not allow the registration of already initiated studies [31]. It should be noted here that the validity of this study was not affected in any way by its non-registration in PROSPERO; all details of the study were fully provided.

### 2.6. Variant Classification in Accordance with Allele Frequency

Variants of interest were classified as very rare (<0.001), rare (from 0.001 to <0.005), low frequency (from 0.005 to 0.05) or common (>0.05) in accordance with their allele frequencies in the studied normal controls, following the definitions of Manolio and colleagues [32].

## 3. Results

### 3.1. Variants Included for Analysis

In the context of the three trypsin-dependent pathway genes, the keyword searches yielded 260 *PRSS1*-related, 391 *SPINK1*-related and 76 *CTRC*-related publications (Supplementary Figure S1). Manual review of these publications in accordance with the criteria specified in Figure 1B identified 17 *PRSS1*-related (Supplementary Table S1), 17 *SPINK1*-related (Supplementary Table S2) and 5 *CTRC*-related (Supplementary Table S3) studies that were then used for analysis. In the end, three distinct *PRSS1* variants, two distinct *SPINK1* variants, three distinct *CTRC* variants as well as one aggregate *CTRC* variant (all rare/very rare pathogenic variants in exons 2, 3 and 7), were retained for analysis (Table 1). It should be noted here that *CTRC* c.180C>T (p.Gly60Gly) was counted as two distinct variants; it was a common variant in the European and American populations [17,33] but rare in the Chinese population [34]. See Supplementary Results, Supplementary Figures S2–S5 and Supplementary Tables S1–S4 for details about how these variants were selected and how their respective OR values were calculated.

There were four other CP genes/loci for which the first disease association report concurrently analyzed ACP patients, NACP patients and normal controls, yielding three distinct informative variants (i.e., *CLDN2* rs7057398, CEL-HYB1 and *CTRB1*-*CTRB2* rs8055167) and one aggregate informative variant (i.e., all *CPA1* variants with apparent activity <20%) (Table 2). A detailed description of these four variants is provided in the Supplementary Results.

In summary, a total of 13 variants were included for analysis.

### 3.2. Evidence Suggesting Extensive G×E Interactions in CP

The paired OR_ACP_ and OR_NACP_ values of the 13 included variants, either newly calculated here or directly taken from the corresponding original reports, are provided in Table 1 and Table 2. Up to seven variants had an OR_ACP_ > OR_NACP_ (5 in Table 1 and 2 in Table 2), suggesting the presence of G×E interactions between the respective variants and alcohol consumption. Three of the seven variants, namely *PRSS1* rs10273639C, *SPINK1* c.101A>G (p.Asn34Ser) and aggregate pathogenic rare/very rare variants in exons 2, 3 and 7 of the *CTRC* gene, were also concurrently analyzed in alcoholic controls and normal controls (See Supplementary Results, Supplementary Figure S6 and Supplementary Table S5 for details about how these variants were selected). None of them showed significant allele difference between the two control datasets (Table 3).

As for the 6 variants that had an OR_ACP_ < OR_NACP_ (4 in Table 1 and 2 in Table 2), we searched for reports in which the age at symptom onset was informative in variant-positive and -negative ACP patients. Four reports were found to be informative with respect to distinct variants. Three of them [35,36,37] reported no significant difference in age at symptom onset between *SPINK1* c.101A>G (p.Asn34Ser)-positive and -negative ACP patients; the remaining one [38] reported no significant difference with respect to two distinct *CPA1* variants, p.Arg254Trp and c.738_761del. However, all these studies were limited by sample size (at most 124 patients were analyzed). We also found two reports that were informative for aggregate variants. Using the Kaplan–Meier model, Zou and colleagues showed that aggregate pathogenic genotypes involving either *PRSS1*, *SPINK1*, *CTRC* and/or *CFTR* (MIM# 602421; encoding cystic fibrosis transmembrane conductance regulator) genes were associated with a significant acceleration in age at symptom onset in ACP (*p* < 0.001; see Supplementary Figure S5 in the original report [34]) (NB, the pathogenic genotypes disproportionately involved *PRSS1* and *SPINK1* variants of strong genetic effect as defined below). The recent Lewis study demonstrated that the median age at symptom onset in ACP patients (defined as an alcohol consumption of >4 drinks per day) with a pathogenic *SPINK1* variant was 39 years whereas that in *SPINK1* variant-negative ACP patients was 45 years, although the difference did not achieve statistical significance [39]. Here, it is pertinent to note that Lewis and colleagues also analyzed a subgroup of CP patients termed “light to moderate drinkers” (defined as an alcohol consumption of ≤4 drinks per day) and found a statistically younger median age at symptom onset in *SPINK1* variant-positive patients as compared to *SPINK1* variant-negative patients (24 vs. 50 years). Details of the pathogenic *SPINK1* variants were not made available in this latter report, but most, if not all, of them should fall into the category of variants of strong genetic effect as defined below. Taken together, these findings supported the presence of G×E interactions in cases wherein a G×E interaction was not immediately evident from a direct comparison of paired OR_ACP_ and OR_NACP_ values.

### 3.3. Inter- and Intra-Variant Comparison of the Paired OR_ACP_ and OR_NACP_ Values Revealed a Dichotomized Genetic Effect

**Figure 2:**
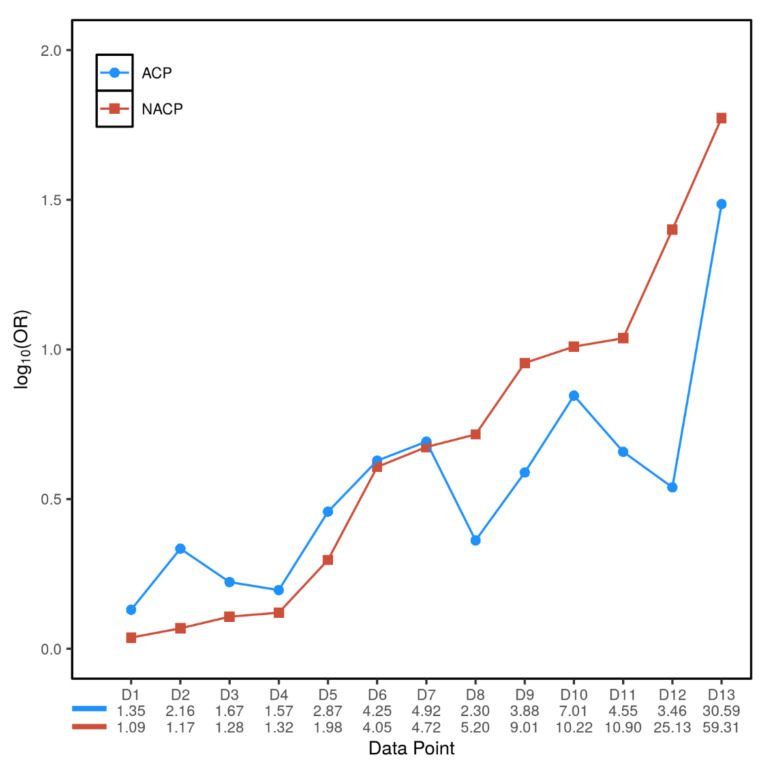
Inter- and intra-variant comparisons of the genetic effects of the included variants on ACP (expressed as OR_ACP_) and NACP (expressed as OR_NACP_). On the *X*-axis, the data points (denoted by D1–D13) were arranged in accordance with the OR_NACP_ values of the variants listed in Table 1 and Table 2, going from the smallest to the largest. ACP, alcoholic chronic pancreatitis; NACP, non-alcoholic chronic pancreatitis; OR_ACP_, odds ratio associated with ACP; OR_NACP_, odds ratio associated with NACP.

To better understand the ground rules for G×E interactions in CP, we next performed an inter- and intra-variant comparison of the paired OR_ACP_ and OR_NACP_ values. Using OR_NACP_ as the baseline genetic effect for comparison, we observed a clear and consistent pattern of genetic effect-dependent dichotomization: thus, variants with OR_NACP_ ranging from 1.09 to 4.72 were invariably found more frequently in ACP than in NACP, whereas variants with OR_NACP_ ranging from 5.20 to 59.31 were invariably found more frequently in NACP than in ACP, with an OR_NACP_ value of ~5 appearing to be the threshold (Figure 2). Based upon this observation, and by reference to the gene effect classifications by Manolio and colleagues [32], we defined here an OR_NACP_ value of <5 as a weak genetic effect and an OR_NACP_ value of ≥5 as a strong genetic effect. In other words, variants with a strong genetic effect were found more frequently in NACP than in ACP, whereas variants with a weak genetic effect were found more frequently in ACP than in NACP.

**Table 2:** Paired OR_ACP_ vs. OR_NACP_ values of included variants from four additional chronic pancreatitis susceptibility genes.

Gene or Locus	Variant	Number of ACP/NACP/Normal Controls	Allele Frequency in ACP	Allele Frequency in NACP	Allele Frequency in Normal Controls	OR_ACP_ (95% CI); *p* Value	OR_NACP_ (95% CI); *p* Value	Source of Data	Data Point Denoted in Figure 2
*CLDN2*	rs12688220		42.7%	32.2%		Not available	Not available	Directly taken from Whitcomb et al. [12]	
	rs7057398					1.57 (1.14–2.18)	1.32 (1.15–1.51)	Directly taken from Derikx et al. [40]	D4
*CPA1*	Aggregate variants with apparent activity <20%	456/944/3938	0.2%	1.5%	0.06%	3.46 (0.67–17.84);	25.13 (9.72–65.02);	Witt et al. [11]; only German data were used.	D12
			(2/912)	(29/1888)	(5/7878)	*p* = 0.34	*p* < 2.2 × 10^−16^		
*CEL*	CEL-HYB1					2.3 (1.2–4.4);	5.2 (3.2–8.5);	Directly taken from Fjeld et al. [41]	D8
						*p* = 0.016	*p* = 1.2 × 10^−11^		
*CTRB1-CTRB2*	rs8055167					1.35 (1.23–1.60)	1.09 (0.82–1.44)	Directly taken from Rosendahl et al. [42]	D1

ACP, alcoholic chronic pancreatitis; CI, confidence interval; NACP, non-alcoholic chronic pancreatitis; OR_ACP_, odds ratio associated with ACP; OR_NACP_, odds ratio associated with NACP.

**Table 3:** Comparison of the risk allele frequencies of the indicated variants between alcoholic controls and normal controls.

Gene	Variant	Number of Alcoholic Controls/Normal Controls	Allele Frequency in Alcoholic Controls	Allele Frequency in Normal Controls	*p* Value	Source of Data
*PRSS1*	rs10273639 (C is the risk allele)	1530/2825	57.7% (1766/3060)	58.5% (3305/5650)	0.85	Derikx et al. [40] (only data from the German subjects were used)
*SPINK1*	c.101A>G (p.Asn34Ser)	305/941	0.49% (3/610)	0.43% (8/1882)	0.91	Meta-analysis of four studies (refer to Figure S6).
*CTRC*	Aggregate pathogenic rare/very rare variants in exons 2, 3 and 7	432/2804	0.46% (4/864)	0.45% (25/5608)	1.00	Rosendahl et al. [10]

## 4. Discussion

Prompted by findings from our recent meta- and re-analyses [13], we have herein embarked on a new analysis of published data with a view to being able to ascertain the scale and basic ground rules of G×E interactions in CP. The premise behind this attempt was that the wealth of genetic and clinical data which has accumulated over the past decades might harbor valuable information that could potentiate epidemiological exploration.

Mindful of the differences in disease definition between different studies in terms of the amount of alcohol consumed and the duration of heavy drinking [34], we opted to use only studies that analyzed variant(s) of interest concurrently in ACP patients, NACP patients and normal controls, with normal controls being employed as a common baseline to allow the simultaneous calculation of OR_ACP_ and OR_NACP_ values (Figure 1). A higher OR_ACP_ than OR_NACP_, which was observed in 7 of the 13 re-analyzed variants, was regarded as an immediate indicator of G×E interactions in CP. Herein, it should be emphasized that we also collated variants that were concurrently analyzed in alcoholic and normal controls in the context of the three trypsin-dependent pathway genes. The respective risk allele frequencies of the three resulting variants, which collectively involved the *PRSS1*, *SPINK1* and *CTRC* genes and whose allele frequencies in normal controls varied from rare to common, were remarkably similar between the two control datasets (Table 3). This allowed us to formally exclude the (albeit inherently unlikely) possibility that a higher OR in ACP than in NACP was actually due to an intrinsic association of the corresponding variant with alcohol consumption. As for variants with a higher OR_NACP_ than OR_ACP_, we also found strong evidence to support the presence of G×E interactions; multiple such variants impacted on age at first pancreatitis symptoms in ACP.

The extensive scale of G×E interactions in CP has received strong support from a mechanistic standpoint. Except for the *PRSS1* c.263G>C (p.Gly208Ala) variant, which may involve mutation-induced misfolding and consequent endoplasmic reticulum stress owing to its negative effect on protein secretion [43], all the distinct *PRSS1*, *SPINK1* and *CTRC* variants (or a *cis*-linked variant in cases such as *PRSS1* rs10273639 [44] and *SPINK1* c.101A>G (p.Asn34Ser) [45]) listed in Table 1 are thought to predispose to CP through their action on the trypsin-dependent pathway of pathology [20]. The recently discovered *CTRB1-CTRB2* inversion polymorphism, tagged by rs8055167 (Table 2), affects protective trypsinogen degradation, thereby ensuring that it should be assigned to the trypsin-dependent pathway [42]. As mentioned earlier, there is a pronounced dosage effect of the rs10273639C risk allele on *PRSS1/PRSS2* mRNA expression in human pancreatic tissue [12,13]; and a synergistic interaction between the risk allele and alcohol consumption has been formally demonstrated [13]. Moreover, and most importantly, mice that express PRSS1^Arg122His^ developed more severe pancreatitis after ethanol feeding [23].

The causal variant underlying the association with CP at the *CLDN2* locus remains to be identified, although the risk genotypes have been linked to ectopic expression of CLDN2 in pancreatic acinar cells [12]. Of the functionally defective *CPA1* variants, the most frequently found variant in NACP patients, c.768C>G (p.Asn256Lys) [11], causes digestive enzyme misfolding and CP in mice [46]. The *CEL-HYB1* allele [41] also appears to cause proteotoxic misfolding owing to missense variants present in the breakpoint junction region of the allele [47]. It should be remembered that the *PRSS1* c.263G>C (p.Gly208Ala) variant may also involve a misfolding pathway that is distinct from the trypsin-dependent pathway [43]. Indeed, the misfolding-dependent pathway is now recognized as an independent mechanism underlying CP [48]. Recently, ethanol feeding was found to accelerate pancreatitis progression in *CPA1* Asn256Lys mutant mice [49].

Despite the heterogeneity in relation both to the genes involved and the risk allele frequencies in normal controls, the re-analyzed variants exhibited a clear and consistent pattern of genetic effect-dependent dichotomization (Figure 2). Here, one may argue that of the seven variants with an OR_ACP_ > OR_NACP_, a statistically significant G×E interaction was demonstrated only for rs10273639C [13] and *CTRC* c.180C>T (p.Gly60Gly) in the context of the LaRush study [33], as evidenced by the non-overlapping 95% CIs (Table 1). By contrast, the other five variants with an OR_ACP_ > OR_NACP_ invariably showed overlapping 95% CIs. Given the clear and consistent pattern of genetic effect-dependent dichotomization exhibited by all analyzed variants (Figure 2), we assume that the absence of statistical significance in most cases was due to a lack of statistical power. Moreover, even in the two cases showing a statistically significant G×E interaction, the absolute differences between the respective OR_ACP_ and OR_NACP_ values were small. Importantly, these consistent observations with nevertheless subtle differences are consistent with a hypothesis about G×E interactions in CP, which was originally postulated by Lankisch and colleagues 20 years ago, in the absence of genetic data; having simply evaluated the impact of alcohol intake on the clinical course of CP, they presciently proposed that common underlying genetic defects exist as the basis of most CP, with environmental factors such as alcohol consumption influencing the expression of the disease [50]. Therefore, it appears that whilst G×E interactions in CP are extensive, they are limited in degree. Additionally, the genetic effect-dependent dichotomization of the re-analyzed variants clearly demonstrate that, as compared to NACP, ACP is more often associated with variants of small genetic effect.

An additional observation merits special mention. Variants having strong effects (i.e., OR_NACP_ of >5) tend to be rare/very rare whereas variants having weak effects (i.e., OR_NACP_ of <5) tend to be common. Consistent with this general trend, *CTRC* c.180C>T (p.Gly60Gly) had an OR_NACP_ of 9.01 when it was a rare variant [34] (in the Chinese population) but had an OR_NACP_ of 1.17 when it was a common variant (in the North American population) [33] (Table 1). This is consistent with our increasing appreciation of ethnic differences in terms of genetic susceptibility to CP [51,52,53] and highlights the importance of the use of ethnically matched controls for genetic association and replication studies.

Our study has several limitations. First and foremost, this study was by no means comprehensive. For example, the *CFTR* gene was not included in the analysis in part due to its extremely high allelic heterogeneity. Secondly, as for the included variants, sample size was quite small in several instances, and this was perhaps the cause of several non-significant associations with ACP or NACP. Thirdly, we would like to reiterate that there were inter-study differences in terms of the ACP and NACP definitions employed although we consider it unlikely that these limitations would have affected the main conclusions drawn owing to the use of paired data in each included study. Finally, it is important to point out that we had to limit our G×E interaction analysis to the ACP context since genetic data on a subgroup of CP patients termed “light to moderate drinkers” [39] have only just begun to emerge. Nonetheless, we surmise that it may be possible to extrapolate from general principles derived from the study of ACP to gene-alcohol interactions in CP as a whole.

## 5. Conclusions

By means of a systematic review, we have uncovered evidence for extensive albeit limited G×E interactions in CP and demonstrated a clear and consistent pattern of genetic effect-dependent dichotomization of the re-analyzed variants. Our findings lend strong support to the hypothesis that alcohol affects the expression of genetically determined CP and highlight a predominant role of variants with a weak genetic effect in the development of ACP. This study also strengthens our appreciation of CP as a multigenic and multifactorial disease and highlights the challenges ahead in terms of variant detection, risk assessment and disease prevention in the age of personalized medicine.

## Figures and Tables

**Table 1:** Paired OR_ACP_ and OR_NACP_ values of the included *PRSS1*, *SPINK1* and *CTRC* variants.

Gene	Variant	Number of ACP/NACP/Normal Controls	Allele Frequency in ACP	Allele Frequency in NACP	Allele Frequency in Normal Controls	OR_ACP_ (95% CI); *p* Value	OR_NACP_ (95% CI); *p* Value	Source of Data	Data Point Denoted in Figure 2
*PRSS1*	rs10273639C/T (C is the risk allele)		65.0%	53.6%		1.67 (1.56–1.78);	1.28 (1.17–1.40);	Directly taken from Herzig et al. [13]	D3
			(3610/5556)	(4341/8094)		*p* < 0.00001	*p* < 0.00001		
	c.365G>A (p.Arg122His)	719/1185/2330	0.7%	0.9%	0.1%	7.01 (1.83–26.77);	10.22 (3.52–29.70);	Meta-analysis of 12 studies (refer to Figure S2)	D10
			10/1438	22/2370	3/4660	*p* = 0.004	*p* < 0.0001		
	c.623G>C (p.Gly208Ala)	206/715/1196	4.4%	4.2%	0.9%	4.92 (2.62–9.26);	4.72 (2.88–7.72); *p* = 9.2 × 10^−11^	Zou et al. [34]	D7
			(18/412)	(60/1430)	(22/2392)	*p* = 3.6 × 10^−7^			
*SPINK1*	c.101A>G (p.Asn34Ser)	1184/1441/4021	3.0%	5.8%	0.6%	4.55 (3.08–6.72);	10.90 (7.56–15.72);	Meta-analysis of 17 studies (refer to Figure S3)	D11
			(72/2368)	(168/2882)	(49/8042)	*p* < 0.00001	*p* < 0.00001		
	c.194+2T>C	206/715/1196	14.3%	24.5%	0.5%	30.59 (16.61–56.34);	59.31 (33.93–103.64);	Zou et al. [34]	D13
			(59/412)	(350/1430)	(13/2392)	*p* < 2.2 × 10^−16^	*p* < 2.2 × 10^−16^		
*CTRC*	Aggregate rare/very rare pathogenic variants in exons 2, 3 and 7	348/758/2804	1.9%	1.8%	0.4%	4.25 (2.16–8.35);	4.05 (2.34–7.00);	Rosendahl et al. [10]	D6
			(13/696)	(27/1516)	(25/5608)	*p* = 2.0 × 10^−5^	*p* = 2.1 × 10^−7^		
	c.760C>T (p.Arg254Trp)	788/1563/4349	0.8%	0.4%	0.3%	2.87 (1.34–6.14);	1.98 (1.03–3.81);	Meta-analysis of four studies (refer to Figure S5)	D5
			(12/1576)	(13/3126)	(22/8698)	*p* = 0.007	*p* = 0.04		
	c.180C>T (p.Gly60Gly)	236/302/1013	20.8%	12.4%	10.8%	2.16 (1.66–2.81);	1.17 (0.89–1.55);	LaRush et al. [33]	D2
			(98/472)	(75/604)	(219/2026)	*p* = 7.7 × 10^−7^	*p* = 0.36		
	c.180C>T (p.Gly60Gly)	206/715/1196	0.5%	1.1%	0.1%	3.88 (0.65–23.32);	9.01 (2.62–30.98);	Zou et al. [34]	D9
			(2/412)	(16/1430)	(3/2392)	*p* = 0.33	*p* = 7.4 × 10^−5^		

ACP, alcoholic chronic pancreatitis; CI, confidence interval; NACP, non-alcoholic chronic pancreatitis; OR_ACP_, odds ratio associated with ACP; OR_NACP_, odds ratio associated with NACP.

## Data Availability

All data relevant to the study are included in the article and uploaded as supplementary information.

## Supplementary Materials

The following are available online at https://www.mdpi.com/article/10.3390/genes12040471/s1, Supplementary Results, Figure S1: Sets and outcomes of the keyword search in “All Fields” of PubMed with respect to *PRSS1*-, *SPINK1*- and *CTRC*-related publications (frozen on 20 November 2020), Figure S2: Meta-analysis of the association between the risk allele of *PRSS1* c.365G>A (p.Arg122His) and alcoholic chronic pancreatitis (A) or non-alcoholic chronic pancreatitis (B), Figure S3: Meta-analysis of the association between the risk allele of *SPINK1* c.101A>G (p.Asn34Ser) and alcoholic chronic pancreatitis (A) or non-alcoholic chronic pancreatitis (B), Figure S4: Funnel plots corresponding to data presented in Supplementary Figure S3, Figure S5: Meta-analysis of the association between the risk allele of *CTRC* c.760C>T (p.Arg254Trp) and alcoholic chronic pancreatitis (A) or non-alcoholic chronic pancreatitis (B), Figure S6: Comparison of the risk allele frequencies of *SPINK1* c.101A>G (p.Asn34Ser) in alcoholic controls (Experimental) and normal controls (Control) by means of meta-analysis, Table S1: *PRSS1*-related studies used for analysis, Table S2: *SPINK1*-related studies used for analysis, Table S3: *CTRC*-related studies used for analysis, Table S4: Pathogenic variants in exons 2, 3 and 7 of the *CTRC* gene in German ACP patients, NACP patients and normal controls, Table S5: Pathogenic variants in exons 2, 3 and 7 of the *CTRC* gene in German alcoholic controls and normal controls.
