# Supplementary material for: Scale and Scope of Gene-Alcohol Interactions in Chronic Pancreatitis: A Systematic Review
Source: Genes (Basel). 2021 Mar 25;12(4):471. doi: 10.3390/genes12040471 (PMC8064432; doi:10.3390/genes12040471)
Supplement: Supplementary file 1 [file genes-12-00471-s001.pdf]

SUPPLEMENTARY RESULTS

Variants Included in the Context of the *PRSS1*, *SPINK1* and *CTRC* Genes

PRSS1

Five of the 17 studies acquired data on the rs10273639-tagging common *PRSS1-PRSS2* haplotype.[1-5] All five studies were included in our recent meta-analysis,[6] in which the risk allele was shown to be significantly associated with both ACP (pooled OR 1.67, 95% CI 1.56–1.78; $P <$ 0.00001) and NACP (pooled OR 1.28, 95% CI 1.17–1.40; $P < 0.00001$) (Table 1).

The remaining 12 studies reported data on the first variant ever reported to cause CP — c.365G>A (p.Arg122His)[7] — in ACP patients, NACP patients and controls,[8-19] although sample sizes were quite small in most studies. Meta-analysis of the corresponding data showed that the risk allele of the c.365G>A variant was significantly associated with both ACP (OR 7.01, 95% CI 1.83–26.77; $P =$ 0.004) and NACP (OR 10.22, 95% CI 3.52–29.70; $P < 0.0001$) (Supplementary Figure S2; Table 1).

Additionally, the Zou study,[19] which screened all exons and exon/intron boundaries of the *PRSS1* gene in 206 ACP patients, 715 ICP patients and 1196 normal controls, reported data on a low-frequency variant, c.623G>C (p.Gly208Ala). The risk allele of this variant was significantly associated with both ACP (OR 4.92, 95% CI 2.62–9.26; $P = 3.6\times10^{-7}$) and NACP (OR 4.72, 95% CI 2.88–7.72; $P = 9.2\times10^{-11}$) (Table 1).

SPINK1

All 17 studies[9-12, 14-26] reported informative data on the extensively studied c.101A>G (p.Asn34Ser) variant.[27-29] Meta-analysis of the 17 studies obtained a pooled OR of 4.55 (95% CI 3.08–6.72; $P < 0.00001$) for ACP and a pooled OR of 10.90 (95% CI 7.56–15.72; $P < 0.00001$) for NACP (Supplementary Figure S3; Table 1). Funnel plot analysis did not reveal asymmetry in either the ACP or NACP context, suggesting the absence of publication bias (Supplementary Figure S4).

The Zou study[19] also reported data on c.194+2T>C, a low-frequency variant in the Chinese population. The risk (C) allele was significantly associated with ACP (OR 30.59, 95% CI 16.61–56.34; $P < 2.2\times10^{-16}$) and NACP (OR 59.31, 95% CI 33.93–103.64; $P < 2.2\times10^{-16}$) (Table 1).

CTRC

Only five studies have reported *CTRC* variant data from ACP patients, NACP patients and normal controls.[19, 26, 30-32] We performed the following analyses:

Firstly, in the pioneering Rosendahl study,[30] which screened all exons of the *CTRC* gene in 758 German NACP patients, only exons 2, 3 and 7 were analyzed in 348 German ACP patients. We therefore performed aggregate analysis with respect to the rare/very rare pathogenic variants in the three commonly analyzed exons. The classification of pathogenic variants was in accordance with the Genetic Risk Factors in Chronic Pancreatitis Database.[33] The aggregate pathogenic alleles (Supplementary Table S4) were significantly associated with ACP (OR 4.25, 95% CI 2.16–8.35; $P =$ 2.0×10^{-5}) and NACP (OR 4.05, 95% CI 2.34–7.00; $P = 2.1\times10^{-7}$) (Table 1).

Secondly, c.760C>T (p.Arg254Trp) in exon 7 was the most frequently detected pathogenic *CTRC* variant in ACP and NACP patients in the Rosendahl study.[30] This variant has also been analyzed concurrently in ACP patients, NACP patients and normal controls in three other studies.[19, 26, 32] We therefore performed a meta-analysis on these four studies (Supplementary Figure S5); the risk allele was found to be associated with a higher risk of ACP (OR 2.87, 95% CI 1.34–6.14; $P = 0.007$) as compared to NACP (OR 1.98, 95% CI 1.03–3.81; $P = 0.04$) (Table 1).

Thirdly, the LaRusch study[31] reported data on c.180C>T (p.Gly60Gly), a common *CTRC* variant in the European population[34] (it should be noted that the frequency of the c.180T allele in non-Finnish

Europeans is 10.4% in accordance with gnomAD data). The risk allele occurred in normal North American controls with a frequency of 10.8% (219/2026)[31] and was associated with a higher risk of ACP (OR 2.16, 95% CI 1.66–2.81; $P = 7.7\times10^{-7}$) as compared to NACP (OR 1.17, 95% CI 0.89–1.55; $P = 0.36$) (Table 1).

Lastly, and perhaps most interestingly, c.180C>T (p.Gly60Gly) turned out to be a rare *CTRC* variant in the Chinese population[19] and was associated with a higher risk for NACP (OR 9.01, 95% CI 2.62–30.98); $P = 7.4\times10^{-5}$) than for ACP (OR 3.88, 95% CI 0.65–23.32); $P = 0.33$) (Table 1).

Variants Included in the Context of Additional CP Susceptibility Genes

There are four other CP genes/loci for which the first disease association report analyzed ACP patients, NACP patients and normal controls. They will be described in chronological order of discovery.

CLDN2

Apart from the rs10273639-tagging common *PRSS1-PRSS2* haplotype, a single nucleotide polymorphism in the X-linked *CLDN2* locus (MIM# 300520; encoding claudin 2), rs12688220, has also been reported, in the first genome-wide association study of CP, to be significantly associated with CP (OR 1.612).[1] The risk allele frequency was higher in ACP than in NACP (42.7% vs. 32.2%) (Table 2), although the corresponding ORs do not appear to have been calculated.[1] The association of the *CLDN2* locus with CP was confirmed in subsequent studies.[2-4] In the first and largest European replication study, rs7057398 and rs12688220 at the X-linked locus were found to be associated with CP and particularly strongly with ACP, but only rs7057398 was found to associate with NACP in female patients.[2] We therefore used the corresponding OR_{ACP} and OR_{NACP} values for rs7057398 in females for re-analysis (Table 2).

CPA1

The ten exons of the *CPA1* gene (MIM# 114850; encoding carboxypeptidase A1) were analyzed in German subjects with ACP, NACP and normal controls. All the functionally deficient variants (defined as having an apparent activity of <20% of wild-type) detected were rare/very rare.[35] We therefore calculated the OR_{ACP} and OR_{NACP} values for the aggregate functionally defective variants, the data being provided in Table 2. None of the subsequent studies analyzed ACP patients.[36-38]

CEL-HYB1

A hybrid allele between the *CEL* gene (MIM# 114840; encoding carboxyl-ester lipase) and its pseudogene (*CELP*), termed CEL-HYB1, was reported to be associated with both ACP and NACP by a European collaborative study.[39] The original OR_{ACP} and OR_{NACP} values, which were expressed in the context of heterozygous carrier frequency, were used for re-analysis (Table 2). CEL-HYB1 was absent from the Chinese, Japanese and Indian populations.[40] A recent Polish study analyzed CEL-HYB1 but only in pediatric CP patients.[41]

CTRB1-CTRB2

An European genome-wide association study identified a common inversion polymorphism in the *CTRB1* (MIM# 118890; encoding chymotrypsinogen B1)-*CTRB2* locus to be associated with ACP and NACP.[42] OR_{ACP} and OR_{NACP} values, both of which were calculated from experimentally determined (not imputed) risk allele frequencies, were available only for the lead rs8055167 variant located in intron 1 of *CTRB1*. Specifically, using samples from the Pan-European Working Group, rs8055167 had an OR_{ACP} of 1.35 and an OR_{NACP} of 1.09. Using a French NACP cohort, rs8055167 had an OR_{NACP}

of 1.52; unfortunately, no corresponding French ACP cohort was analyzed.[42] We therefore used data obtained from the samples of the Pan-European Working Group for re-analysis (Table 2). In an independent (and the only) replication study, the risk allele did not contribute to disease risk variation in the Chinese population due to near fixation of the disease allele.[43]

Informative CP Risk Variants in the *PRSS1*, *SPINK1* and *CTRC* Genes between Alcoholic Controls and Normal Controls

PRSS1

Four reports described *PRSS1* variant data in both alcoholic controls and normal controls.[2, 8, 44, 45] Three of them were informative for c.365G>A (p.Arg122His) and c.86A>T (p.Asn29Ile),[8, 44, 45] the first two variants reported to cause CP.[7, 46] Together, however, these three studies analyzed a total of only 95 alcoholic controls and 77 normal controls. It was therefore not at all surprising that neither variant was found in these controls because both variants fall into the category of 'very rare variants' [c.365G>A has an allele frequency of 0.00001062 whereas c.86A>T is absent from the Genome Aggregation Database (gnomAD; https://gnomad.broadinstitute.org/).[47]

The fourth study analyzed the rs10273639C/T-tagged common *PRSS1-PRSS2* haplotype in 887 German subjects with alcohol dependence, 643 German subjects with alcoholic liver cirrhosis and 2825 German normal controls.[2] Since the frequencies of the risk (C) allele were remarkably similar between subjects with alcohol dependence and subjects with alcoholic liver cirrhosis (57.4% (1018/1774) vs. 58.2% (748/1286); $P = 0.85$), we combined them into a single alcoholic control group. The frequency of the rs10273639 risk (C) allele in this single alcoholic control group was not however significantly different from that in the normal control group (57.7% (1766/3060) vs. 58.5% (3305/5650); $P = 0.85$) (Table 3).

SPINK1

Four reports that used samples from four different populations described *SPINK1* variant data in both alcoholic controls and normal controls.[25, 26, 44, 48] All involved the most extensively studied c.101A>G (p.Asn34Ser) variant,[27] analyzing a total of 305 alcoholic controls and 941 normal controls. Meta-analysis showed that the variant did not differ significantly between the two control groups in terms of its risk allele frequency distribution (0.49% (3/610) vs. 0.43% (8/1882); $P = 0.91$) (Supplementary Figure S6; Table 3).

CTRC

Only two reports described *CTRC* variant data in both alcoholic and normal controls. The first study sequenced exons 2, 3 and 7 of the *CTRC* gene in both German alcoholic controls and normal controls.[30] Since all pathogenic variants (in accordance with the classifications in the Genetic Risk Factors in Chronic Pancreatitis database[33]) fell into either the category of rare variants or the category of very rare variants, they were subjected to an aggregate analysis (Supplementary Table S5). The frequency of the aggregate risk alleles in the alcoholic controls was not significantly different from that in the normal controls (0.46% (4/864) vs. 0.45% (25/5608); $P = 1.00$) (Table 3).

The second study was limited for two reasons. First, it only sought the rare c.760C>T (p.Arg254Trp) variant located in exon 7.[26] Second, it only analyzed 110 alcoholic controls and 297 normal controls. The c.760C>T variant was found only once in each control group.

PRSS1-related publications

Search	Actions	Details	Query	Results
#5	•••	>	Search: **#1 or #2 or #3 or #4** Sort by: **Most Recent**	260
#4	•••	>	Search: **trypsinogen alcohol pancreatitis** Sort by: **Most Recent**	227
#3	•••	>	Search: **trypsinogen alcoholic pancreatitis** Sort by: **Most Recent**	96
#2	•••	>	Search: **prss1 alcohol pancreatitis** Sort by: **Most Recent**	90
#1	•••	>	Search: **prss1 alcoholic pancreatitis** Sort by: **Most Recent**	54

SPINK1-related publications

Search	Actions	Details	Query	Results
#7	•••	>	Search: **#1 or #2 or #3 or #4 or #5 or #6** Sort by: **Most Recent**	391
#6	•••	>	Search: **trypsin inhibitor alcohol pancreatitis** Sort by: **Most Recent**	358
#5	•••	>	Search: **trypsin inhibitor alcoholic pancreatitis** Sort by: **Most Recent**	92
#4	•••	>	Search: **PSTI alcohol pancreatitis** Sort by: **Most Recent**	22
#3	•••	>	Search: **PSTI alcoholic pancreatitis** Sort by: **Most Recent**	14
#2	•••	>	Search: **spink1 alcohol pancreatitis** Sort by: **Most Recent**	122
#1	•••	>	Search: **SPINK1 alcoholic pancreatitis** Sort by: **Most Recent**	75

CTRC-related publications

Search	Actions	Details	Query	Results
#5	•••	>	Search: **#1 or #2 or #3 or #4** Sort by: **Most Recent**	76
#4	•••	>	Search: **chymotrypsinogen alcohol pancreatitis** Sort by: **Most Recent**	58
#3	•••	>	Search: **chymotrypsinogen alcoholic pancreatitis** Sort by: **Most Recent**	12
#2	•••	>	Search: **CTRC alcohol pancreatitis** Sort by: **Most Recent**	23
#1	•••	>	Search: **CTRC alcoholic pancreatitis** Sort by: **Most Recent**	12

Supplementary Figure S1. Sets and outcomes of the keyword search in “All Fields” of PubMed with respect to *PRSS1*-, *SPINK1*- and *CTRC*-related publications (frozen on 20 November 2020).

A

Study or Subgroup	Experimental Events	Experimental Total	Control Events	Control Total	Weight	Odds Ratio M-H, Fixed, 95% CI
Bernardino et al. 2003	0	128	0	400		Not estimable
Chandak et al. 2004	0	82	0	580		Not estimable
Drenth et al. 2002	0	144	0	240		Not estimable
Gasiorowska et al. 2011	7	66	0	92	19.8%	23.32 [1.31, 415.91]
Lee et al. 2004	0	94	0	38		Not estimable
Liu et al. 2008	0	70	0	240		Not estimable
Liu et al. 2017	0	48	0	200		Not estimable
Midha et al. 2010	0	84	0	200		Not estimable
Mora et al. 2009	0	156	0	168		Not estimable
O'Reilly et al. 2001	1	72	0	40	33.4%	1.70 [0.07, 42.69]
Sisman et al. 2015	0	82	0	70		Not estimable
Zou et al. 2018	2	412	3	2392	46.8%	3.88 [0.65, 23.32]
**Total (95% CI)**		**1438**		**4660**	**100.0%**	**7.01 [1.83, 26.77]**
Total events	10		3			

Heterogeneity: Chi² = 1.83, df = 2 (P = 0.40); I² = 0%
Test for overall effect: Z = 2.85 (P = 0.004)

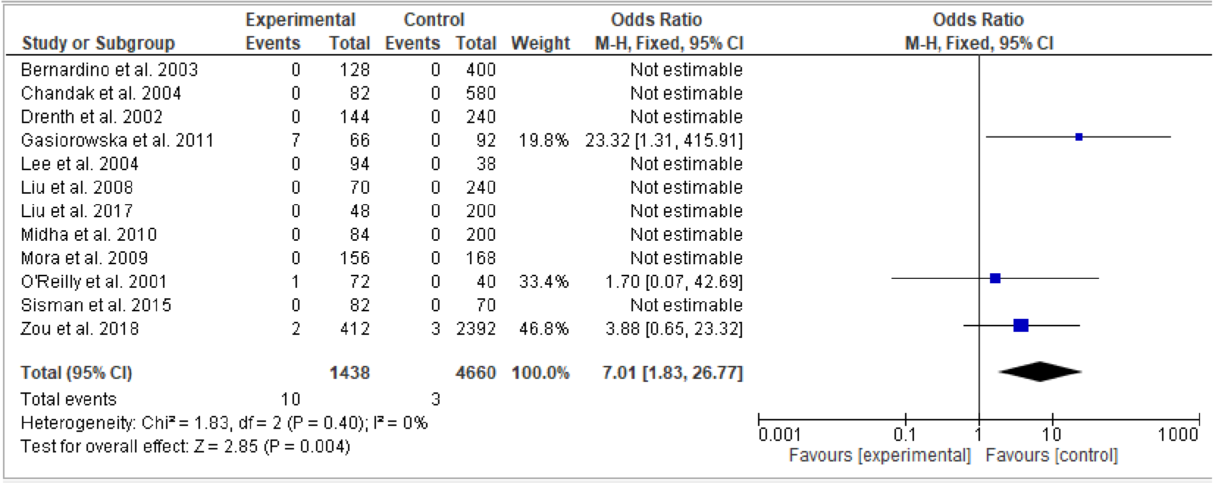

B

Study or Subgroup	Experimental Events	Experimental Total	Control Events	Control Total	Weight	Odds Ratio M-H, Fixed, 95% CI
Bernardino et al. 2003	0	32	0	400		Not estimable
Chandak et al. 2004	0	240	0	580		Not estimable
Drenth et al. 2002	0	48	0	240		Not estimable
Gasiorowska et al. 2011	2	28	0	92	7.6%	17.45 [0.81, 374.81]
Lee et al. 2004	0	44	0	38		Not estimable
Liu et al. 2008	0	34	0	240		Not estimable
Liu et al. 2017	1	152	0	200	15.0%	3.97 [0.16, 98.14]
Midha et al. 2010	0	226	0	200		Not estimable
Mora et al. 2009	0	32	0	168		Not estimable
O'Reilly et al. 2001	0	28	0	40		Not estimable
Sisman et al. 2015	0	76	0	70		Not estimable
Zou et al. 2018	19	1430	3	2392	77.4%	10.72 [3.17, 36.30]
**Total (95% CI)**		**2370**		**4660**	**100.0%**	**10.22 [3.52, 29.70]**
Total events	22		3			

Heterogeneity: Chi² = 0.46, df = 2 (P = 0.80); I² = 0%
Test for overall effect: Z = 4.27 (P < 0.0001)

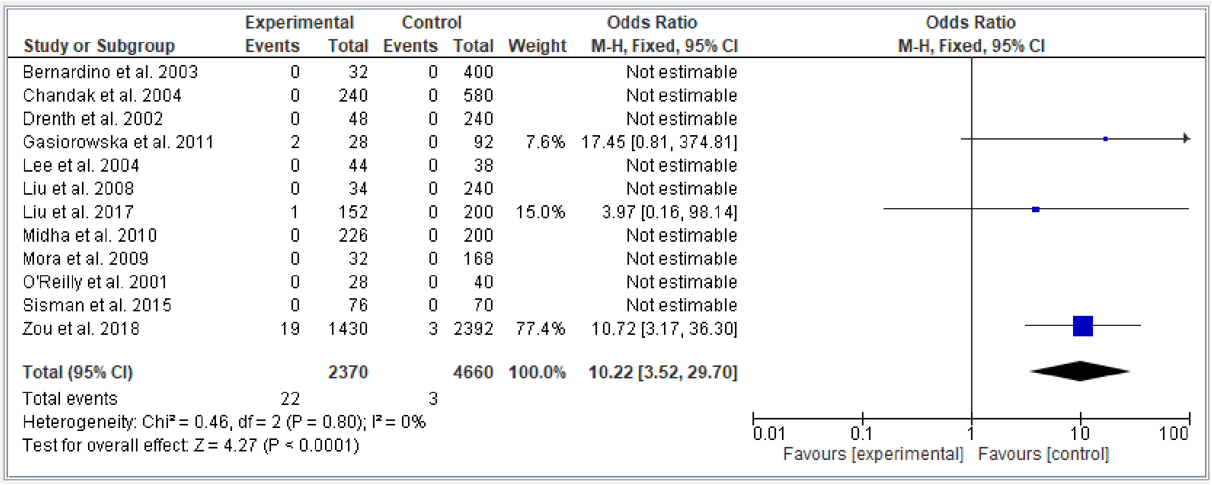

Supplementary Figure S2. Meta-analysis of the association between the risk allele of *PRSS1* c.365G>A (p.Arg122His) and alcoholic chronic pancreatitis (**A**) or non-alcoholic chronic pancreatitis (**B**). Experimental, patient.

A

Study or Subgroup	Patient Events	Patient Total	Control Events	Control Total	Weight	Odds Ratio M-H, Fixed, 95% CI
Bernardino et al. 2003	0	128	0	400		Not estimable
Chandak et al. 2004	11	82	8	580	7.8%	11.08 [4.31, 28.46]
Cichoz-Lach et al. 2012	10	134	2	124	8.7%	4.92 [1.06, 22.92]
da Costa et al. 2016	3	220	1	594	2.4%	8.20 [0.85, 79.23]
Diaconu et al. 2009	4	160	1	192	4.0%	4.90 [0.54, 44.27]
Drenth et al. 2002	5	144	2	240	6.6%	4.28 [0.82, 22.36]
Gasiorowska et al. 2011	9	66	4	92	13.1%	3.47 [1.02, 11.81]
Lee et al. 2004	1	94	0	38	3.2%	1.24 [0.05, 31.00]
Lempinen et al. 2005	10	174	12	918	16.4%	4.60 [1.96, 10.83]
Liu et al. 2017	1	48	0	200	0.9%	12.66 [0.51, 315.73]
Midha et al. 2010	7	84	4	200	9.9%	4.45 [1.27, 15.65]
Mora et al. 2009	3	156	0	168	2.1%	7.68 [0.39, 149.96]
Schneider et al. 2003	2	64	3	380	3.8%	4.05 [0.66, 24.75]
Shimosegawa et al. 2008	0	186	2	1054	3.4%	1.13 [0.05, 23.60]
Sisman et al. 2015	0	82	0	70		Not estimable
Threadgold et al. 2002	4	134	5	400	11.1%	2.43 [0.64, 9.19]
Zou et al. 2018	2	412	5	2392	6.6%	2.33 [0.45, 12.04]
**Total (95% CI)**		**2368**		**8042**	**100.0%**	**4.55 [3.08, 6.72]**
Total events	72		49			

Heterogeneity: Chi² = 7.34, df = 14 (P = 0.92); I² = 0%

Test for overall effect: Z = 7.60 (P < 0.00001)

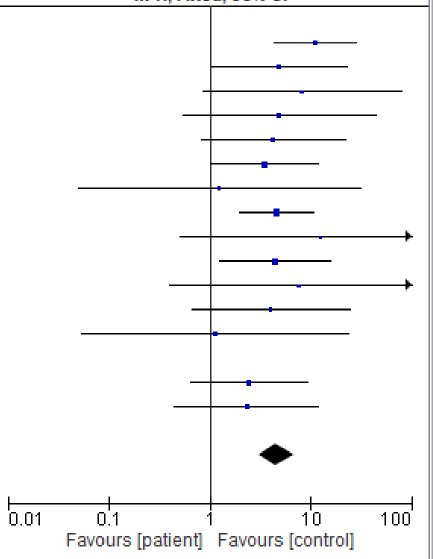

B

Study or Subgroup	Patient Events	Patient Total	Control Events	Control Total	Weight	Odds Ratio M-H, Fixed, 95% CI
Bernardino et al. 2003	0	32	0	400		Not estimable
Chandak et al. 2004	45	240	8	580	19.2%	16.50 [7.64, 35.61]
Cichoz-Lach et al. 2012	2	70	2	124	7.1%	1.79 [0.25, 13.02]
da Costa et al. 2016	2	76	1	594	1.1%	16.03 [1.44, 178.91]
Diaconu et al. 2009	0	20	1	192	1.4%	3.11 [0.12, 78.94]
Drenth et al. 2002	5	48	2	240	3.0%	13.84 [2.60, 73.63]
Gasiorowska et al. 2011	6	28	4	92	7.4%	6.00 [1.56, 23.11]
Lee et al. 2004	0	44	0	38		Not estimable
Lempinen et al. 2005	5	40	12	918	4.4%	10.79 [3.60, 32.29]
Liu et al. 2017	0	152	0	200		Not estimable
Midha et al. 2010	56	226	4	200	16.1%	16.14 [5.73, 45.44]
Mora et al. 2009	3	32	0	168	0.7%	39.98 [2.01, 794.21]
Schneider et al. 2003	5	78	3	380	4.8%	8.61 [2.01, 36.81]
Shimosegawa et al. 2008	7	116	2	1054	1.9%	33.78 [6.93, 164.62]
Sisman et al. 2015	0	76	0	70		Not estimable
Threadgold et al. 2002	13	174	5	400	14.2%	6.38 [2.24, 18.18]
Zou et al. 2018	19	1430	5	2392	18.6%	6.43 [2.40, 17.25]
**Total (95% CI)**		**2882**		**8042**	**100.0%**	**10.90 [7.56, 15.72]**
Total events	168		49			

Heterogeneity: Chi² = 11.25, df = 12 (P = 0.51); I² = 0%

Test for overall effect: Z = 12.80 (P < 0.00001)

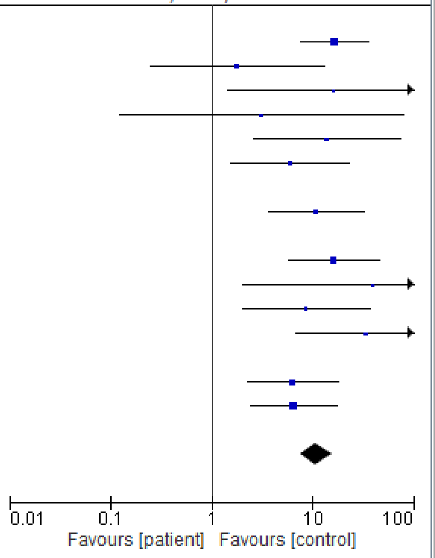

Supplementary Figure S3. Meta-analysis of the association between the risk allele of *SPINK1* c.101A>G (p.Asn34Ser) and alcoholic chronic pancreatitis (**A**) or non-alcoholic chronic pancreatitis (**B**).

A

B

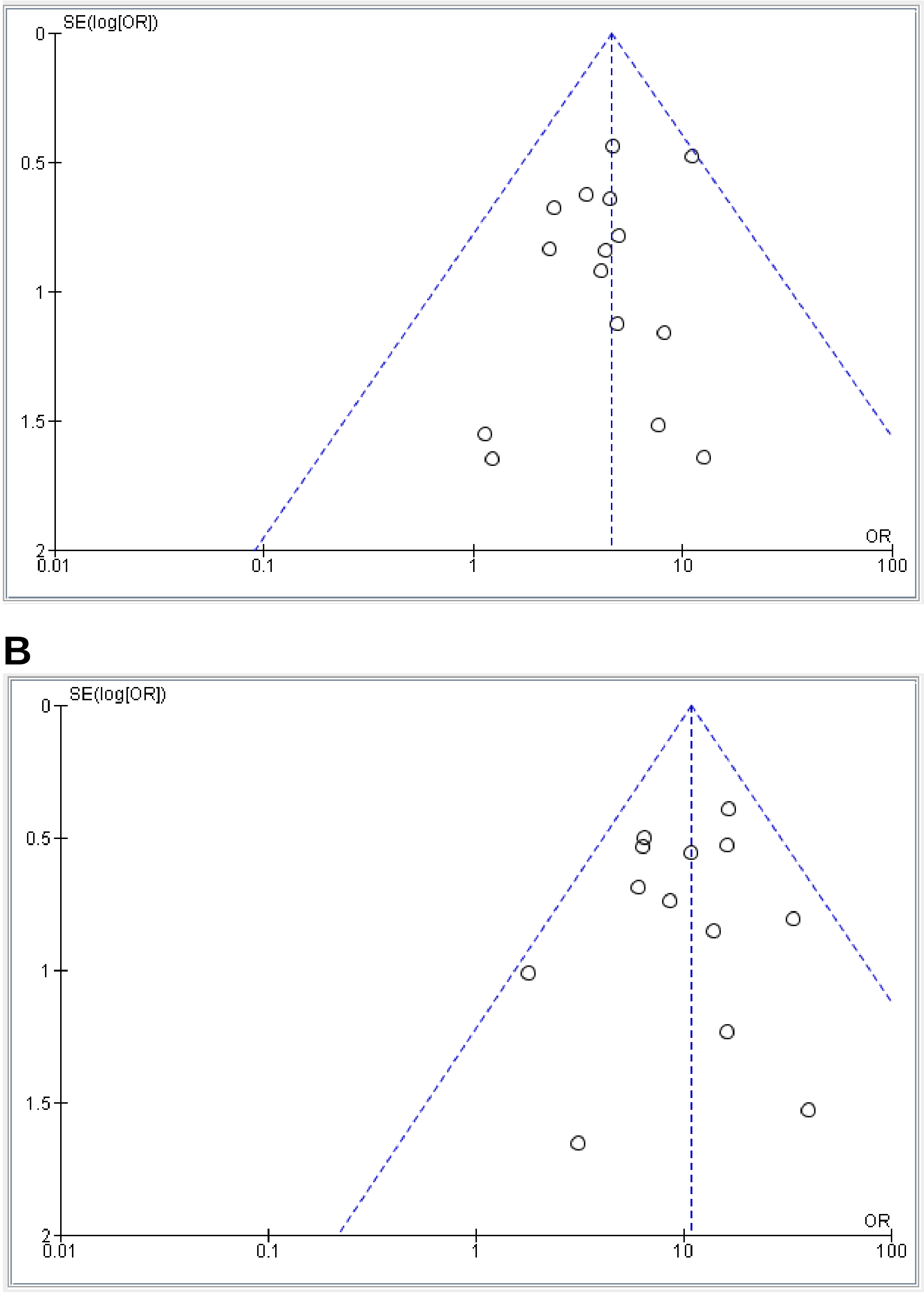

Supplementary Figure S4. Funnel plots corresponding to data presented in Supplementary Figure S3.

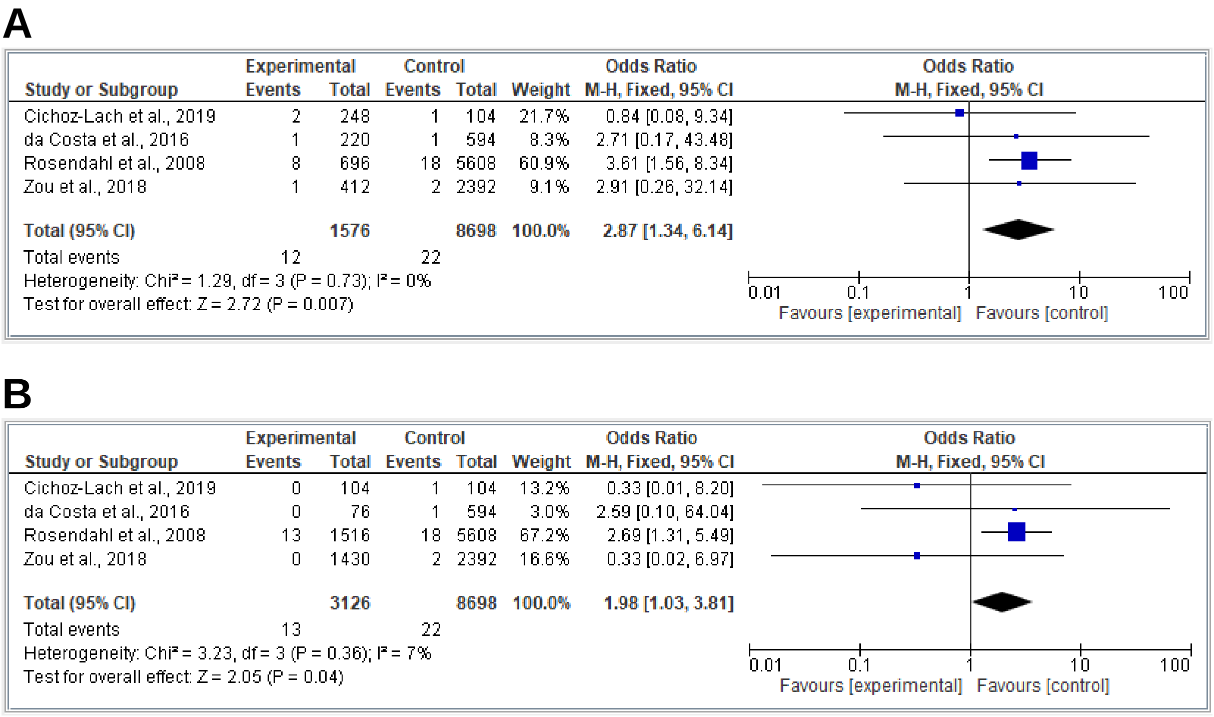

Supplementary Figure S5. Meta-analysis of the association between the risk allele of *CTRC* c.760C>T (p.Arg254Trp) and alcoholic chronic pancreatitis (**A**) or non-alcoholic chronic pancreatitis (**B**). Experimental, patient.

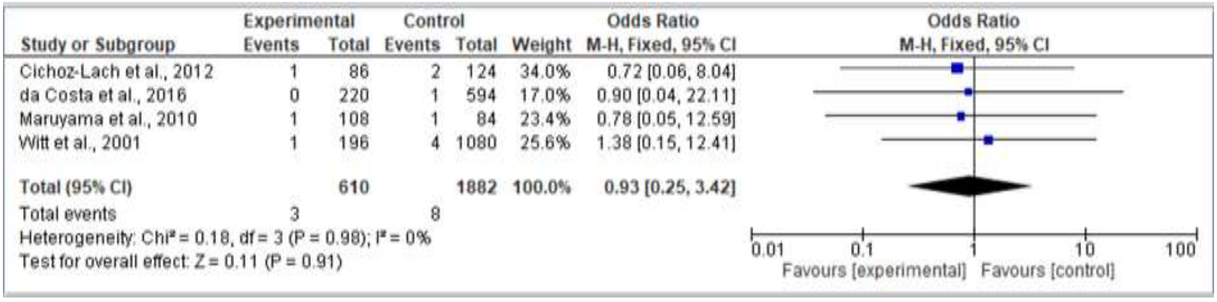

Supplementary Figure S6. Comparison of the risk allele frequencies of *SPINK1* c.101A>G (p.Asn34Ser) in alcoholic controls (Experimental) and normal controls (Control) by means of meta-analysis.

Supplementary Table S1. *PRSS1*-related studies used for analysis

**Reference**	**Number of ACP patients**	**Number of NACP patients**	**Number of normal controls**
Hegyi E, Tóth AZ, Vincze Á, Szentesi A, Hegyi P, Sahin-Tóth M. Alcohol-dependent effect of *PRSS1-PRSS2* haplotype in chronic pancreatitis. *Gut*. 2020;69(9):1-2. doi:10.1136/gutjnl-2019-319729	120	103	296
Zou WB, Tang XY, Zhou DZ, et al. *SPINK1*, *PRSS1*, *CTRC*, and *CFTR* genotypes influence disease onset and clinical outcomes in chronic pancreatitis. *Clin Transl Gastroenterol*. 2018;9(11):204. Published 2018 Nov 12. doi:10.1038/s41424-018-0069-5	206	715	1196
Liu X, Tu M, Dai X, et al. *PRSS1* and *SPINK1* Mutations in alcoholic and idiopathic chronic pancreatitis. *J Nanosci Nanotechnol*. 2017;17(4):2358-2362. doi:10.1166/jnn.2017.12626	24	76	100
Giri AK, Midha S, Banerjee P, et al. Common variants in *CLDN2* and *MORC4* genes confer disease susceptibility in patients with chronic pancreatitis. *PLoS One*. 2016;11(1):e0147345. Published 2016 Jan 28. doi:10.1371/journal.pone.0147345	85	434	1288
Masamune A, Nakano E, Hamada S, Kakuta Y, Kume K, Shimosegawa T. Common variants at *PRSS1-PRSS2* and *CLDN2-MORC4* loci associate with chronic pancreatitis in Japan. *Gut*. 2015;64(8):1345-1346. doi:10.1136/gutjnl-2015-309802	272	197	480
Şişman G, Tuğcu M, Ayla K, Sebati Ö, Şentürk H. Mutation analysis of *PRSS1*, *SPINK1* and *CFTR* gene in patients with alcoholic and idiopathic chronic pancreatitis: A single center study. *Turk J Gastroenterol*. 2015;26(2):176-180. doi:10.5152/tjg.2015.4287	41	38	35
Derikx MH, Kovacs P, Scholz M, et al. Polymorphisms at *PRSS1-PRSS2* and *CLDN2-MORC4* loci associate with alcoholic and non-alcoholic chronic pancreatitis in a European replication study. *Gut*. 2015;64(9):1426-1433. doi:10.1136/gutjnl-2014-307453	1854	1192	2825
Whitcomb DC, LaRusch J, Krasinskas AM, et al. Common genetic variants in the *CLDN2* and *PRSS1-PRSS2* loci alter risk for alcohol-related and sporadic pancreatitis. *Nat Genet*. 2012;44(12):1349-1354. doi:10.1038/ng.2466	447	1129	8029
Gasiorowska A, Talar-Wojnarowska R, Czupryniak L, et al. The prevalence of cationic trypsinogen (*PRSS1*) and serine protease inhibitor, Kazal type 1 (*SPINK1*) gene mutations in Polish patients with alcoholic and idiopathic chronic pancreatitis. *Dig Dis Sci*. 2011;56(3):894-901. doi:10.1007/s10620-010-1349-4	33	14	46

Midha S, Khajuria R, Shastri S, Kabra M, Garg PK. Idiopathic chronic pancreatitis in India: phenotypic characterisation and strong genetic susceptibility due to *SPINK1* and *CFTR* gene mutations. *Gut.* 2010;59(6):800-807. doi:10.1136/gut.2009.191239	42	113	100
Mora J, Comas L, Ripoll E, et al. Genetic mutations in a Spanish population with chronic pancreatitis. *Pancreatology*. 2009;9(5):644-651. doi:10.1159/000181177	78	16	84
Liu QC, Gao F, Ou QS, et al. Novel mutation and polymorphism of *PRSS1* gene in the Chinese patients with hereditary pancreatitis and chronic pancreatitis. *Chin Med J (Engl).* 2008;121(2):108-111.	35	17	120
Lee, K. H., Yoon, W. J., Ryu, J. K., Kim, Y. T., Yoon, Y. B., & Kim, C. Y. [Mutations of *SPINK1* and *PRSS1* gene in Korean patients with chronic pancreatitis]. *Korean J Gastroenterol*. 2004 Aug;44(2):93-8.	47	22	19
Chandak GR, Idris MM, Reddy DN, et al. Absence of *PRSS1* mutations and association of *SPINK1* trypsin inhibitor mutations in hereditary and non-hereditary chronic pancreatitis. *Gut.* 2004;53(5):723-728. doi:10.1136/gut.2003.026526	41	120	290
Bernardino AL, Guarita DR, Mott CB, et al. *CFTR*, *PRSS1* and *SPINK1* mutations in the development of pancreatitis in Brazilian patients. *JOP*. 2003;4(5):169-177.	64	16	200
Drenth JP, te Morsche R, Jansen JB. Mutations in serine protease inhibitor Kazal type 1 are strongly associated with chronic pancreatitis. *Gut.* 2002;50(5):687-692. doi:10.1136/gut.50.5.687	72	24	120
O'Reilly DA, Yang BM, Creighton JE, Demaine AG, Kingsnorth AN. Mutations of the cationic trypsinogen gene in hereditary and non-hereditary pancreatitis. *Digestion.* 2001;64(1):54-60. doi:10.1159/000048839	36	14	20

Supplementary Table S2. *SPINK1*-related studies used for analysis

Reference	Number of ACP patients	Number of NACP patients	Number of normal controls
Zou WB, Tang XY, Zhou DZ, et al. *SPINK1, PRSS1, CTRC*, and *CFTR* genotypes influence disease onset and clinical outcomes in chronic pancreatitis. *Clin Transl Gastroenterol*. 2018;9(11):204. Published 2018 Nov 12. doi:10.1038/s41424-018-0069-5	206	715	1196
Liu X, Tu M, Dai X, et al. *PRSS1* and *SPINK1* mutations in alcoholic and idiopathic chronic pancreatitis. *J Nanosci Nanotechnol*. 2017;17(4):2358-2362. doi:10.1166/jnn.2017.12626	24	76	100
da Costa MZ, Pires JG, Nasser PD, et al. Frequency of tabagism and N34S and P55S mutations of serine peptidase inhibitor, Kazal Type 1 (*SPINK1*) and R254W mutation of chymotrypsin C (*CTRC*) in patients with chronic pancreatitis and controls. *Pancreas*. 2016;45(9):1330-1335. doi:10.1097/MPA.0000000000000650	110	38	297
Şişman G, Tuğcu M, Ayla K, Sebati Ö, Şentürk H. Mutation analysis of *PRSS1, SPINK1* and *CFTR* gene in patients with alcoholic and idiopathic chronic pancreatitis: A single center study. *Turk J Gastroenterol*. 2015;26(2):176-180. doi:10.5152/tjg.2015.4287	41	38	35
Cichoż-Lach H, Michalak M, Lis E, et al. The N34S mutation of the *SPINK1* gene and alcoholic chronic pancreatitis. *Pol Arch Med Wewn.* 2012;122(6):277-283. doi:10.20452/pamw.1293	67	35	62
Gasiorowska A, Talar-Wojnarowska R, Czupryniak L, et al. The prevalence of cationic trypsinogen (*PRSS1*) and serine protease inhibitor, Kazal type 1 (*SPINK1*) gene mutations in Polish patients with alcoholic and idiopathic chronic pancreatitis. *Dig Dis Sci*. 2011;56(3):894-901. doi:10.1007/s10620-010-1349-4	33	14	46
Midha S, Khajuria R, Shastri S, Kabra M, Garg PK. Idiopathic chronic pancreatitis in India: phenotypic characterisation and strong genetic susceptibility due to *SPINK1* and *CFTR* gene mutations. *Gut.* 2010;59(6):800-807. doi:10.1136/gut.2009.191239	42	113	100
Diaconu BL, Ciobanu L, Mocan T, et al. Investigation of the *SPINK1* N34S mutation in Romanian patients with alcoholic chronic pancreatitis. A clinical analysis based on the criteria of the M-ANNHEIM classification. *J Gastrointestin Liver Dis.* 2009;18(2):143-150.	80	10	96
Mora J, Comas L, Ripoll E, et al. Genetic mutations in a Spanish population with chronic pancreatitis. *Pancreatology.* 2009;9(5):644-651. doi:10.1159/000181177	78	16	84

Shimosegawa T, Kume K, Masamune A. *SPINK1*, *ADH2*, and *ALDH2* gene variants and alcoholic chronic pancreatitis in Japan. *J Gastroenterol Hepatol*. 2008;23 Suppl 1:S82-S86. doi:10.1111/j.1440-1746.2007.05291.x	93	58	527
Lempinen M, Paju A, Kemppainen E, et al. Mutations N34S and P55S of the *SPINK1* gene in patients with chronic pancreatitis or pancreatic cancer and in healthy subjects: a report from Finland. *Scand J Gastroenterol.* 2005;40(2):225-230. doi:10.1080/00365520510011560	87	20	459
Chandak GR, Idris MM, Reddy DN, et al. Absence of *PRSS1* mutations and association of *SPINK1* trypsin inhibitor mutations in hereditary and non-hereditary chronic pancreatitis. *Gut.* 2004;53(5):723-728. doi:10.1136/gut.2003.026526	41	120	290
Lee, K. H., Yoon, W. J., Ryu, J. K., Kim, Y. T., Yoon, Y. B., & Kim, C. Y. [Mutations of *SPINK1* and *PRSS1* gene in Korean patients with chronic pancreatitis]. *Korean J Gastroenterol*. 2004 Aug;44(2):93-8.	47	22	19
Bernardino AL, Guarita DR, Mott CB, et al. *CFTR, PRSS1* and *SPINK1* mutations in the development of pancreatitis in Brazilian patients. *JOP*. 2003;4(5):169-177.	64	16	200
Schneider A, Pfützer RH, Barmada MM, Slivka A, Martin J, Whitcomb DC. Limited contribution of the *SPINK1* N34S mutation to the risk and severity of alcoholic chronic pancreatitis: a report from the United States. *Dig Dis Sci*. 2003;48(6):1110-1115. doi:10.1023/a:1023768829772	32	39	190
Drenth JP, te Morsche R, Jansen JB. Mutations in serine protease inhibitor Kazal type 1 are strongly associated with chronic pancreatitis. *Gut.* 2002;50(5):687-692. doi:10.1136/gut.50.5.687	72	24	120
Threadgold J, Greenhalf W, Ellis I, et al. The N34S mutation of *SPINK1* (PSTI) is associated with a familial pattern of idiopathic chronic pancreatitis but does not cause the disease. *Gut.* 2002;50(5):675-681. doi:10.1136/gut.50.5.675	67	87	200

Supplementary Table S3. *CTRC*-related studies used for analysis

Reference	Number of ACP patients	Number of NACP patients	Number of normal controls
Zou WB, Tang XY, Zhou DZ, et al. *SPINK1*, *PRSS1*, *CTRC*, and *CFTR* genotypes influence disease onset and clinical outcomes in chronic pancreatitis. *Clin Transl Gastroenterol*. 2018;9(11):204. Published 2018 Nov 12. doi:10.1038/s41424-018-0069-5	206	715	1196
Cichoż-Lach H, Michalak-Wojnowska M, Lis-Janczarek E, Wojcierowski J, Hydzik M. Do *CTRC* mutations affect the development of alcoholic chronic pancreatitis and its course among Poles: Preliminary study. *Adv Clin Exp Med*. 2019;28(3):307-312. doi:10.17219/acem/76130	124	52	52
da Costa MZ, Pires JG, Nasser PD, et al. Frequency of tabagism and N34S and P55S mutations of serine peptidase inhibitor, Kazal Type 1 (*SPINK1*) and R254W mutation of chymotrypsin C (CTRC) in Patients With Chronic Pancreatitis and Controls. *Pancreas*. 2016;45(9):1330-1335. doi:10.1097/MPA.0000000000000650	110	38	297
LaRusch J, Lozano-Leon A, Stello K, et al. The common chymotrypsinogen C (*CTRC*) variant G60G (C.180T) increases risk of chronic pancreatitis but not recurrent acute pancreatitis in a North American population. *Clin Transl Gastroenterol*. 2015;6(1):e68. Published 2015 Jan 8. doi:10.1038/ctg.2014.13	206	715	1013
Rosendahl J, Witt H, Szmola R, et al. Chymotrypsin C (*CTRC*) variants that diminish activity or secretion are associated with chronic pancreatitis. *Nat Genet*. 2008;40(1):78-82. doi:10.1038/ng.2007.44	348	758	2804

Supplementary Table S4. Pathogenic variants in exons 2, 3 and 7 of the *CTRC* gene in German ACP patients, NACP patients and normal controls*

Exon	Variant	Number of ACP patients/NACP patients/normal controls	Allele frequency in ACP patients	Allele frequency in NACP patients	Allele frequency in normal controls
3	c.143A>G (p.Gln48Arg)	348/758/2804[a]	0/696	0/1516	1/5608
7	c.649G>A (p.Gly217Ser)	348/758/2804	0/696	2/1516	1/5608
7	c.649G>C (p.Gly217Arg)	348/758/2804	1/696	0/1516	0/5608
7	c.659T>G (p.Leu220Arg)	348/758/2804	0/696	0/1516	1/5608
7	c.703G>A (p.Val235Ile)	348/758/2804	1/696	1/1516	1/5608
7	c.738_761del (p.Lys247_Arg254del)	348/758/2804	2/696	11/1516	3/5608
7	c.746C>T (p.Pro249Leu)	348/758/2804	1/696	0/1516	0/5608
7	c.760C>T (p.Arg254Trp)	348/758/2804	8/696	13/1516	18/5608
	Aggregate data	348/758/2804	13/696	27/1516	25/5608

*Variant data from Rosendahl et al. (2008).[30]

[a]The total number of normal controls analyzed was 2689 in the original report. It was changed to 2804 for the purpose of aggregate analysis.

Abbreviations: ACP, alcoholic chronic pancreatitis; NACP, non-alcoholic chronic pancreatitis.

Supplementary Table S5. Pathogenic variants in exons 2, 3 and 7 of the *CTRC* gene in German alcoholic controls and normal controls*

Exon	Variant	Number of alcoholic controls/normal controls	Allele frequency in alcoholic controls	Allele frequency in normal controls
3	c.143A>G (p.Gln48Arg)	432/2804[a]	0/864	1/5608
7	c.649G>A (p.Gly217Ser)	432/2804	0/864	1/5608
7	c.649G>C (p.Gly217Arg)	432/2804	0/864	0/5608
7	c.659T>G (p.Leu220Arg)	432/2804	0/864	1/5608
7	c.703G>A (p.Val235Ile)	432/2804	1/864	1/5608
7	738_761del (p.Lys247_Arg254del)	432/2804	1/864	3/5608
7	c.746C>T (p.Pro249Leu)	432/2804	0/864	0/5608
7	c.760C>T (p.Arg254Trp)	432/2804	2/864	18/5608
	Aggregate data	432/2804	4/864	25/5608

*Variant data from Rosendahl et al. (2008).[30]

[a]The total number of normal controls analyzed was 2689 in the original report. It was changed to 2804 for the purpose of aggregate analysis.

Supplementary References

1. Whitcomb DC, LaRusch J, Krasinskas AM, et al. Common genetic variants in the *CLDN2* and *PRSS1-PRSS2* loci alter risk for alcohol-related and sporadic pancreatitis. *Nat Genet* 2012; 44: 1349-54.
2. Derikx MH, Kovacs P, Scholz M, et al. Polymorphisms at *PRSS1-PRSS2* and *CLDN2-MORC4* loci associate with alcoholic and non-alcoholic chronic pancreatitis in a European replication study. *Gut* 2015; 64: 1426-33.
3. Masamune A, Nakano E, Hamada S, et al. Common variants at *PRSS1-PRSS2* and *CLDN2-MORC4* loci associate with chronic pancreatitis in Japan. *Gut* 2015; 64: 1345-6.
4. Giri AK, Midha S, Banerjee P, et al. Common variants in *CLDN2* and *MORC4* genes confer disease susceptibility in patients with chronic pancreatitis. *PLoS One* 2016; 11: e0147345.
5. Hegyi E, Toth AZ, Vincze A, et al. Alcohol-dependent effect of *PRSS1-PRSS2* haplotype in chronic pancreatitis. *Gut* 2020; 69: 1713-15.
6. Herzig AF, Genin E, Cooper DN, et al. Role of the common *PRSS1-PRSS2* haplotype in alcoholic and non-alcoholic chronic pancreatitis: Meta- and re-analyses. *Genes (Basel)* 2020; 11: 1349.
7. Whitcomb DC, Gorry MC, Preston RA, et al. Hereditary pancreatitis is caused by a mutation in the cationic trypsinogen gene. *Nat Genet* 1996; 14: 141-5.
8. O'Reilly DA, Yang BM, Creighton JE, et al. Mutations of the cationic trypsinogen gene in hereditary and non-hereditary pancreatitis. *Digestion* 2001; 64: 54-60.
9. Drenth JP, te Morsche R, Jansen JB. Mutations in serine protease inhibitor Kazal type 1 are strongly associated with chronic pancreatitis. *Gut* 2002; 50: 687-92.
10. Bernardino AL, Guarita DR, Mott CB, et al. *CFTR*, *PRSS1* and *SPINK1* mutations in the development of pancreatitis in Brazilian patients. *JOP* 2003; 4: 169-77.
11. Chandak GR, Idris MM, Reddy DN, et al. Absence of *PRSS1* mutations and association of *SPINK1* trypsin inhibitor mutations in hereditary and non-hereditary chronic pancreatitis. *Gut* 2004; 53: 723-8.
12. Lee KH, Yoon WJ, Ryu JK, et al. [Mutations of *SPINK1* and *PRSS1* gene in Korean patients with chronic pancreatitis]. *Korean J Gastroenterol* 2004; 44: 93-8.
13. Liu QC, Gao F, Ou QS, et al. Novel mutation and polymorphism of *PRSS1* gene in the Chinese patients with hereditary pancreatitis and chronic pancreatitis. *Chin Med J (Engl)* 2008; 121: 108-11.
14. Mora J, Comas L, Ripoll E, et al. Genetic mutations in a Spanish population with chronic pancreatitis. *Pancreatology* 2009; 9: 644-51.
15. Midha S, Khajuria R, Shastri S, et al. Idiopathic chronic pancreatitis in India: phenotypic characterisation and strong genetic susceptibility due to *SPINK1* and *CFTR* gene mutations. *Gut* 2010; 59: 800-7.
16. Gasiorowska A, Talar-Wojnarowska R, Czupryniak L, et al. The prevalence of cationic trypsinogen (*PRSS1*) and serine protease inhibitor, Kazal type 1 (*SPINK1*) gene mutations in Polish patients with alcoholic and idiopathic chronic pancreatitis. *Dig Dis Sci* 2011; 56: 894-901.
17. Sisman G, Tugcu M, Ayla K, et al. Mutation analysis of *PRSS1*, *SPINK1* and *CFTR* gene in patients with alcoholic and idiopathic chronic pancreatitis: A single center study. *Turk J Gastroenterol* 2015; 26: 176-80.
18. Liu X, Tu M, Dai X, et al. *PRSS1* and *SPINK1* mutations in alcoholic and idiopathic chronic pancreatitis. *J Nanosci Nanotechnol* 2017; 17: 2358-362.
19. Zou WB, Tang XY, Zhou DZ, et al. *SPINK1*, *PRSS1*, *CTRC*, and *CFTR* genotypes influence disease onset and clinical outcomes in chronic pancreatitis. *Clin Transl Gastroenterol* 2018; 9: 204.
20. Threadgold J, Greenhalf W, Ellis I, et al. The N34S mutation of *SPINK1* (*PSTI*) is associated with a familial pattern of idiopathic chronic pancreatitis but does not cause the disease. *Gut* 2002; 50: 675-81.
21. Schneider A, Pfutzer RH, Barmada MM, et al. Limited contribution of the *SPINK1* N34S mutation to the risk and severity of alcoholic chronic pancreatitis: a report from the United States. *Dig Dis Sci* 2003; 48: 1110-5.

22. Lempinen M, Paju A, Kemppainen E, et al. Mutations N34S and P55S of the *SPINK1* gene in patients with chronic pancreatitis or pancreatic cancer and in healthy subjects: a report from Finland. *Scand J Gastroenterol* 2005; 40: 225-30.
23. Shimosegawa T, Kume K, Masamune A. *SPINK1*, *ADH2*, and *ALDH2* gene variants and alcoholic chronic pancreatitis in Japan. *J Gastroenterol Hepatol* 2008; 23 Suppl 1: S82-6.
24. Diaconu BL, Ciobanu L, Mocan T, et al. Investigation of the *SPINK1* N34S mutation in Romanian patients with alcoholic chronic pancreatitis. A clinical analysis based on the criteria of the M-ANNHEIM classification. *J Gastrointestin Liver Dis* 2009; 18: 143-50.
25. Cichoz-Lach H, Michalak M, Lis E, et al. The N34S mutation of the *SPINK1* gene and alcoholic chronic pancreatitis. *Pol Arch Med Wewn* 2012; 122: 277-83.
26. da Costa MZ, Pires JG, Nasser PD, et al. Frequency of tabagism and N34S and P55S mutations of serine peptidase inhibitor, Kazal Type 1 (*SPINK1*) and *R254W* mutation of chymotrypsin C (*CTRC*) in patients with chronic pancreatitis and controls. *Pancreas* 2016; 45: 1330-5.
27. Witt H, Luck W, Hennies HC, et al. Mutations in the gene encoding the serine protease inhibitor, Kazal type 1 are associated with chronic pancreatitis. *Nat Genet* 2000; 25: 213-6.
28. Aoun E, Chang CC, Greer JB, et al. Pathways to injury in chronic pancreatitis: decoding the role of the high-risk *SPINK1* N34S haplotype using meta-analysis. *PLoS One* 2008; 3: e2003.
29. Di Leo M, Bianco M, Zuppardo RA, et al. Meta-analysis of the impact of *SPINK1* p.N34S gene variation in Caucasic patients with chronic pancreatitis. An update. *Dig Liver Dis* 2017; 49: 847-53.
30. Rosendahl J, Witt H, Szmola R, et al. Chymotrypsin C (*CTRC*) variants that diminish activity or secretion are associated with chronic pancreatitis. *Nat Genet* 2008; 40: 78-82.
31. LaRusch J, Lozano-Leon A, Stello K, et al. The common chymotrypsinogen C (*CTRC*) variant G60G (c.180T) increases risk of chronic pancreatitis but not recurrent acute pancreatitis in a north american population. *Clin Transl Gastroenterol* 2015; 6: e68.
32. Cichoz-Lach H, Michalak-Wojnowska M, Lis-Janczarek E, et al. Do *CTRC* mutations affect the development of alcoholic chronic pancreatitis and its course among Poles: Preliminary study. *Adv Clin Exp Med* 2019; 28: 307-12.
33. Genetic Risk Factors in Chronic Pancreatitis. http://www.pancreasgenetics.org/index.php.
34. Masson E, Chen JM, Scotet V, et al. Association of rare chymotrypsinogen C (*CTRC*) gene variations in patients with idiopathic chronic pancreatitis. *Hum Genet* 2008; 123: 83-91.
35. Witt H, Beer S, Rosendahl J, et al. Variants in *CPA1* are strongly associated with early onset chronic pancreatitis. *Nat Genet* 2013; 45: 1216-20.
36. Wu H, Zhou DZ, Berki D, et al. No significant enrichment of rare functionally defective *CPA1* variants in a large Chinese idiopathic chronic pancreatitis cohort. *Hum Mutat* 2017; 38: 959-63.
37. Kujko AA, Berki DM, Oracz G, et al. A novel p.Ser282Pro CPA1 variant is associated with autosomal dominant hereditary pancreatitis. *Gut* 2017; 66: 1728-30.
38. Nemeth BC, Orekhova A, Zhang W, et al. Novel p.K374E variant of CPA1 causes misfolding-induced hereditary pancreatitis with autosomal dominant inheritance. *Gut* 2020; 69: 790-92.
39. Fjeld K, Weiss FU, Lasher D, et al. A recombined allele of the lipase gene *CEL* and its pseudogene *CELP* confers susceptibility to chronic pancreatitis. *Nat Genet* 2015; 47: 518-22.
40. Zou WB, Boulling A, Masamune A, et al. No association between CEL-HYB hybrid allele and chronic pancreatitis in Asian populations. *Gastroenterology* 2016; 150: 1558-60 e5.
41. Oracz G, Kujko AA, Fjeld K, et al. The hybrid allele 1 of carboxyl-ester lipase (CEL-HYB1) in Polish pediatric patients with chronic pancreatitis. *Pancreatology* 2019; 19: 531-34.
42. Rosendahl J, Kirsten H, Hegyi E, et al. Genome-wide association study identifies inversion in the *CTRB1-CTRB2* locus to modify risk for alcoholic and non-alcoholic chronic pancreatitis. *Gut* 2018; 67: 1855-63.
43. Tang XY, Zou WB, Masson E, et al. The *CTRB1-CTRB2* risk allele for chronic pancreatitis discovered in European populations does not contribute to disease risk variation in the Chinese population due to near allele fixation. *Gut* 2018; 67: 1368-69.

44. Maruyama K, Harada S, Yokoyama A, et al. Association analyses of genetic polymorphisms of *GSTM1*, *GSTT1*, *NQO1*, *NAT2*, *LPL, PRSS1, PSTI*, and *CFTR* with chronic alcoholic pancreatitis in Japan. *Alcohol Clin Exp Res* 2010; 34 Suppl 1: S34-8.
45. Teich N, Mossner J, Keim V. Screening for mutations of the cationic trypsinogen gene: are they of relevance in chronic alcoholic pancreatitis? *Gut* 1999; 44: 413-6.
46. Gorry MC, Gabbaizedeh D, Furey W, et al. Mutations in the cationic trypsinogen gene are associated with recurrent acute and chronic pancreatitis. *Gastroenterology* 1997; 113: 1063-8.
47. Lek M, Karczewski KJ, Minikel EV, et al. Analysis of protein-coding genetic variation in 60,706 humans. *Nature* 2016; 536: 285-91.
48. Witt H, Luck W, Becker M, et al. Mutation in the *SPINK1* trypsin inhibitor gene, alcohol use, and chronic pancreatitis. *JAMA* 2001; 285: 2716-7.
